# Implementing a complex rehabilitation intervention in a stroke trial: a qualitative process evaluation of AVERT

**DOI:** 10.1186/s12874-016-0156-9

**Published:** 2016-05-10

**Authors:** Julie A Luker, Louise E Craig, Leanne Bennett, Fiona Ellery, Peter Langhorne, Olivia Wu, Julie Bernhardt

**Affiliations:** Centre for Research Excellence in Stroke Rehabilitation and Brain Recovery, Florey Institute of Neuroscience and Mental Health, University of Melbourne, Melbourne, Australia; International Centre for Allied Health Evidence, University of South Australia, Adelaide, Australia; Nursing Research Institute, Australian Catholic University, Sydney, Australia; Institute of Cardiovascular & Medical Sciences, University of Glasgow, Glasgow, Scotland UK; Institute of Health and Wellbeing, University of Glasgow, Glasgow, Scotland UK; Latrobe University, Melbourne, Australia

**Keywords:** Stroke, Rehabilitation, Clinical trials, Clinical research protocol, Qualitative research, Implementation

## Abstract

**Background:**

The implementation of multidisciplinary stroke rehabilitation interventions is challenging, even when the intervention is evidence-based. Very little is known about the implementation of complex interventions in rehabilitation clinical trials.

The aim of study was to better understand how the implementation of a rehabilitation intervention in a clinical trial within acute stroke units is experienced by the staff involved. This qualitative process evaluation was part of a large Phase III stroke rehabilitation trial (AVERT).

**Methods:**

A descriptive qualitative approach was used. We purposively sampled 53 allied health and nursing staff from 19 acute stroke units in Australia, New Zealand and Scotland. Semi-structured interviews were conducted by phone, voice-internet, or face to face. Digitally recorded interviews were transcribed and analysed by two researchers using rigorous thematic analysis.

**Results:**

Our analysis uncovered ten important themes that provide insight into the challenges of implementing complex new rehabilitation practices within complex care settings, plus factors and strategies that assisted implementation. Themes were grouped into three main categories: staff experience of implementing the trial intervention, barriers to implementation, and overcoming the barriers. Participation in the trial was challenging but had personal rewards and improved teamwork at some sites. Over the years that the trial ran some staff perceived a change in usual care. Barriers to trial implementation at some sites included poor teamwork, inadequate staffing, various organisational barriers, staff attitudes and beliefs, and patient-related barriers. Participants described successful implementation strategies that were built on interdisciplinary teamwork, education and strong leadership to ‘get staff on board’, and developing different ways of working.

**Conclusions:**

The AVERT stroke rehabilitation trial required commitment to deliver an intervention that needed strong collaboration between nurses and physiotherapists and was different to current care models. This qualitative process evaluation contributes unique insights into factors that may be critical to successful trials teams, and as AVERT was a pragmatic trial, success factors to delivering complex intervention in clinical practice.

**Trial registration:**

AVERT registered with Australian New Zealand Clinical Trials Registry ACTRN12606000185561.

**Electronic supplementary material:**

The online version of this article (doi:10.1186/s12874-016-0156-9) contains supplementary material, which is available to authorized users.

## Background

The implementation of new clinical practices within hospital settings is difficult even when the targeted practices have a sound evidence base [1]. Efforts to implement evidence-based practices in acute stroke units (ASUs) have had mixed success despite systematic national initiatives [2, 3]. The evidence for effective implementation strategies for ASU settings is underdeveloped.

Clinician behaviour change is particularly challenging when the new clinical intervention is unproven, as occurs when conducting pragmatic trials within ASUs [4]. Stroke rehabilitation interventions are complex interventions [5] and the study of such interventions is considered more difficult than that of a conventional drug intervention. Campbell and colleagues [5] define complex interventions as being “built up from a number of components, which may act both independently and interdependently.” Such intervention delivery often involves staff from a range of disciplines within a dynamic healthcare setting, as occurred in the A Very Early Rehabilitation Trial (AVERT) [6]. Published evidence to guide implementation in such trial situations is rare. While principles can be applied from the broader implementation science field, many of the issues faced within ASUs and within trials are unique.

The influence of context on the implementation of complex interventions is important, whether applying this to clinical practice improvements [1], or to conducting research trials [4, 7]. Contextual influences may include the hospital environment, systems of work, management and existing policies. Frequently clinical staff have to deliver the trial intervention within their existing role and multidisciplinary team. Some clinicians’ limited experience of delivering an intervention in a trial setting may pose further challenges. The requirement of these staff to alter their usual practice during the trial brings with it the complication and unpredictability of human behaviour change [8, 9].

Factors which may have influenced the success or failure of implementation, and information about any enabling strategies should be collected prospectively alongside the main evaluation [10]. This can help reduce the unacceptable waste identified in many clinical trials [11–13]. Process evaluations can provide data about the implementation of the intervention during the lifespan of the trial as well as assist the interpretation of outcome data [14, 15]. Although still uncommon, recommended multi-method process evaluations in stroke rehabilitation research are beginning to appear. For example, recent trials of early communication therapy [16], patient-led therapy [17], and carer training for inpatients after stroke [15].

The recently conducted pragmatic, multicentre, rehabilitation trial (AVERT) provides an opportunity to improve our understanding of the implementation of complex rehabilitation trial interventions in acute settings.

### The AVERT intervention

This Phase III trial was registered with the Australian New Zealand Clinical Trials Registry, number ACTRN12606000185561 and the protocol is available online [18].

In summary AVERT was a parallel group single blind randomised controlled trial conducted in 56 ASUs in five countries, recruiting 2104 acute stroke patients between 2006–2014. Eligible patients were randomised, stratified by site and severity, to receive usual stroke unit care alone or very early mobilisation (VEM) in addition to usual care for up to 14 days after stroke or until discharge from the ASU. The VEM intervention was guided by a detailed protocol and involved beginning therapy within 24 h of stroke onset, frequent training (at least three additional sessions each day), and therapy focus on task specific sitting, standing and walking activities. The functional ability of the patient determined the dose of therapy received and was adjusted in line with recovery. VEM was provided by ASU physiotherapy and nursing staff who opted to be involved in the trial and received training in VEM delivery. Compliance to protocol was very high and significant dose differences between the arms of the trial (usual care and VEM) were confirmed [6].

The aim of this qualitative process evaluation is to understand the implementation of an acute stroke rehabilitation trial intervention as perceived by the healthcare professionals delivering it. Having insight into the experiential knowledge and perspectives of these staff is crucial to develop the implementation evidence-base for acute stroke rehabilitation (trial) interventions.

## Methods

This study uses a descriptive qualitative methodology [19, 20].

Collaborations between AVERT researchers in Australia, Scotland and New Zealand enabled this study. Ethical approvals were obtained in all three countries: Human Research Ethics Committee, Austin Health, Australia (LNR/13/Austin/169); Research Office, Auckland District Health Board, Auckland, New Zealand (ADHB/A+ 6208); Scotland A Research Ethics Committee, NHS Greater Glasgow and Clyde, Scotland (10/S1001/52).

### Authors’ relationship with participants

As chief investigator (JB) and trial manager (FE) had relationships with all participants and so were not involved with data collection or analysis. JL had been the main investigator at one ASU, thus did not collect data at that site. The principle analyst for first stage coding (LB) had no other involvement with the trial or participants. In Scotland recruitment, data collection and initial analysis was undertaken by LC as part of her PhD. The raw Scottish data that were relevant to our research questions (i.e. related to implementing the VEM protocol) were re-analysed along with the southern hemisphere data by JL.

## Study sample

Using purposive sampling staff at participating AVERT sites, who had been involved with the trial, were invited to participate. In Australian and New Zealand recruitment was overseen by the trial manager who invited participation via a monthly investigator newsletter and email correspondence. Potential participants in Scotland were approached by nurse unit managers or by LC. Of the 72 staff who initially expressed interest in participating, six did not go on to consent for unknown reasons, and one moved overseas after consenting and was not contactable for interview.

We obtained informed consent from 36 Australian, five New Zealand and 12 Scottish participants (*n* = 53 total). The sample comprised of 33 physiotherapists (PT), 18 nurses (NS), one PT assistant, and one speech pathologist from 14 ASUs in Australia, one New Zealand and four Scottish ASUs.

## Data collection & management

All qualitative data were collected and analysed before the results of the trial were known in order to minimise bias in researchers’ data collection and analysis or in interviewees’ responses. We conducted semi-structured interviews using interview guides that allowed for additional questioning or probes if interesting information arose (see Table 1). Interviews focused on the implementation of the VEM protocol (the rehabilitation intervention), rather than administrative and trial management aspects.

**Table 1:** Interview Guide example used for Australia & New Zealand

1	What was your role with AVERT?
	Think about actually providing very early mobilisation intervention (VEM):
2	Tell me about your experience of providing very early mobilisation (VEM) in your acute stroke unit.
3	Are there factors that negatively impact on the ability to provide VEM in your unit? Have you found any ways to get around these barriers?
4	Are there factors that you think positively facilitate the provision of VEM in your unit?
5	Are there any strategies or tools that help with VEM decisions? (Such as deciding whether VEM will be safe or appropriate for new patients)
6	Do you have any strategies to ensure patients were provided with sufficient VEM? (frequency)
7	What is your personal opinion of VEM at this point in time?
	Thinking back to the start of AVERT at your stroke unit:
8	How would you describe team work in your setting, or the ability of the stroke team to problem-solve and implement practice improvements?
9	Describe the way your work-place went about implementing VEM in the beginning. Who had any influence on the implementation of VEM, and describe how?
10	At this time the results of AVERT are *still unknown*. However *if* VEM was proven to significantly improve stroke outcomes, *and if* it became recommended standard practice:
	What would you recommend to other acute stroke units wanting to implement VEM practices?
11	Who should be involved in organising the implementation of new VEM practices?
12	Who should be involved in providing the VEM intervention?

All Australian and New Zealand interviews were conducted between Jan–Aug 2014 at a time convenient to participants, one-to-one by telephone or voice-internet (JL, LB). Scottish interviews were conducted from Dec 2010 to May 2011, in participants’ workplace face-to-face (two participants) or within three focus groups (10 participants) (LC). Although repeat interviews were not conducted, two participants had two-part interviews conducted due to interruptions. Telephone interviews commonly ran for about 30 min (range 11–61mins); Scottish face-to-face interviews averaged 59 min (35–95mins).

Interviews were audio recorded, transcribed verbatim by LC and a professional service, and loaded onto software for data management (NVivo 10, QRS International Pty Ltd). Field notes captured basic context and contact information. We returned transcripts to participants for member checking if they wanted, but no subsequent comments or corrections were received. Participants and ASUs were de-identified prior to analysis.

Examples from the dataset supporting the conclusions of this article is included in Additional file 1.

## Analysis

Our rigorous descriptive qualitative methods used thematic analysis to explore the experiences and perspectives of staff involved in the trial. This approach uses low-inference interpretation to present a rich description of experiences or processes in everyday language, and is especially relevant to multi-methods health research such as AVERT [19, 20].

We used a staged process to inductively code the data and identifying themes. Each stage involved independent consideration by two or more researchers, discussions and consensus. Initially we became familiar with the data through repeated listening and reading of the interviews. Secondly we coded the transcripts allocating codes to small sections of meaning; LB working on NVivo and JL coding on hard copies, leading to 1828 initial codes. Through an iterative process of constant comparison across transcripts, we then grouped the codes into logical and meaningful clusters in a hierarchical tree structure, forming categories, descriptive themes and subthemes (see Table 2). To ensure we stayed close to the data, interview quotations that aligned to each theme (see Additional file 1) were threaded through the results.

**Table 2:** Final coding tree

Themes		Interviews^a^	Sub-themes
	Category 1: Staff experience of implementing the trial intervention		
1	Extra work but rewarding	27	
2	Team practice changes	24	
			Changes to usual care
	Category 2: Barriers to intervention implementation		
3	Team challenges	19	`
4	Staffing challenges	37	
5	Organisational or workplace barriers	28	
			The acute model and culture
			Barriers to ASU access:
			Competing priorities
			Physical environment barriers
6	Staff attitudes and beliefs	32	
			Not ‘on board’
			Beliefs about roles and capabilities
			Beliefs about consequences
7	Patients’ barriers	35	
			Acuity, instability and complexity
			Severity of stroke
			Fatigue
			Family anxiety
	Category 3: Overcoming implementation barriers		
8	Teamwork central to success	43	
			Communication and coordination
9	Getting staff ‘on board’	35	
			Staff education and training
			Leadership for change
10	Working differently	29	
			‘This is what we do here’
			Shifting control
			Staffing model changes
			Dealing with fatigue

^a^number of interviews containing data supporting the theme

A level of data saturation was reached. The Scottish data were analysed last and aligned with the 10 major themes found from Australian/New Zealand data. However there were some new findings at sub-theme level.

## Results

Ten emergent themes, each with subthemes, were grouped into three main categories: staff experience of implementing the trial intervention, barriers to implementation, and strategies used to overcome the barriers (Table 2).

Interviewed staff described the challenges of participation in the trial and how their site undertook implementation of the VEM protocol.

### Staff experience of implementing the trial intervention

#### Extra work but rewarding

There was widespread hope that the trial may benefit stroke patient outcomes. Most staff described considerable challenges in managing the additional work associated with the trial. These challenges did have positive consequences for some individuals as described by one physiotherapist:

*It was time-consuming. There were times when I would need to do extra …see patients in lunch time…It added interest to the workday and defined a new challenge at work. So I found it quite enjoyable [PT]*

#### Team practices changes

Although it was not an aim of the trial, several staff reported that it had a positive impact on teamwork at their site. A stated objective of intervention delivery was for nurses and physiotherapists to work together to achieve the required intervention dose. Having common interdisciplinary trial goals had improved team organisation, communication and planning, and increased interdisciplinary practices.

Nurses and physiotherapists noticed changes in their professional roles during the trial and many felt that participation had raised their profile and reinvigorated their clinical practice. Nurses delivered more mobilisations into their daily patient care than previously. Increased hands-on therapy time was noted by physiotherapists and many reported this, and the challenge of finding strategies to increase mobilisation, as rewarding.

#### Changes to usual practice

Over the years that the trial ran, some Australian and New Zealand staff perceived a change in usual care, with all stroke patients tending to be mobilised earlier and more intensively after stroke.

*…what we considered to be our standard care had changed dramatically, from the time of commencing AVERT, to the time of when we eventually finished. [NS]*

This was not a universal perception, with several interviewees reporting that mobilising patients early was their standard practice.

### Barriers to intervention implementation

Staff reported encountering barriers that either affected general implementation of the trial at their site, or barriers more specific to the VEM intervention. VEM starting very early after stroke, and/or being frequently applied to patients, were potentially problematic.

#### Team challenges

Implementation difficulties were experienced at sites that appeared to lack established interdisciplinary teamwork. One PT reported that nurses and physiotherapists working separately to provide VEM at their site created difficulties:

*I think if you had …a whole ward team that was on board and …really interested and really proactive about it, then it would make it significantly easier to do. That has been a difficulty for us [PT]*

Staff reported that the success of VEM implementation was very dependent on effective, enthusiastic on-site team leadership. Trial involvement often became more difficult or even unsustainable when leaders left. Some teams also experienced difficulties providing VEM when there was no nurse leader and a PT tried to lead nurses. For example:

*…she was a nurse …I did find it challenging when she wasn’t there to get the nurses on board [PT]*

#### Staffing challenges

Both physiotherapists and nurses reported inadequate staffing levels made it difficult to consistently implement VEM on top of high existing workloads. Competing demands, such as staff having clinical commitments with other units, made it particularly difficult for physiotherapists. The competing needs of non-trial patients caused a tension for some staff who were conscious of providing an equitable service for all.

Experienced, trained staff were essential for successful VEM provision and a lack of adequately trained nurses was a common barrier. Reduced out-of-hours staffing caused additional difficulties with weekend and night nurses tending to have less training, confidence or priority for providing VEM. Many sites also reported difficulties caused by staff turnover, with experienced staff leaving and replaced with untrained staff and/or those who were less committed to the trial.

*…maintaining staff has been a significant barrier…nurses move on, timeframes for recruitment of your nursing staff, and also pregnancies and all those sorts of things. [NS]*

#### Organisational or workplace barriers

*The acute model and culture:* The fast paced, high acuity, discharge driven culture of acute hospitals, where rehabilitation has low priority, was frequently reported as a barrier to VEM implementation as explained by one nurse:

*…the acute nurses on the stroke unit don't necessarily have that…culture of getting them up, getting them moving [NS]*

Upon admission patients underwent multiple assessments, tests and procedures on and off the ward. This made it difficult to access patients to commence and continue VEM.

*Barriers to ASU access:* Particular problems for commencing VEM within the early timeframe were patient delays in getting to hospital after stroke. Once in the hospital further delays were experienced while patients waited in ED for a bed in the ASU or as outlies in other wards. The provision of VEM was very difficult when patients were not in the ASU. Where stroke patients were housed in wards with other diagnostic groups, problems arose from a dilution of staff with stroke-specific skills and interests.

*Competing priorities:* Difficulties were experienced when there were competing organisational priorities such as discharge pressures, hospital accreditation work, and ward transfer policies. For example:

*…if the AVERT patient is the most stable …they would be transferred to another ward…I'd have to run and see them…but even trickier for the nurses [PT]*

Some sites were affected by organisational changes that could derail trial participation, such as disbanding the ASU which led to withdrawal from the trial for one site. Restrictive manual handling policies and inflexible mealtimes and rest periods also limited the provision of VEM at some sites. Conflicting clinical protocols, such as resting after thrombolysis, were occasionally problematic but usually resolved through negotiation with medical staff. Within ASUs problems resulted when trial-trained nurses were not rostered to recruited patients.

While staff reported strong support from the external AVERT management team, a few sites experienced problematic resistance or lack of encouragement from hospital management, medical staff or from PT department heads. The reasons for managerial resistance were unclear but staff thought conflicting clinical priorities, lack of interest in research and revenue implications (in private hospitals) were influences.

*Physical environment barriers:* Sites varied considerably in their set-up and for some, environmental issues made VEM difficult. Lack of equipment such as supportive chairs, patient lifters and lifter slings were problematic. The ward environment or access to a gym were only raised as problems at two Scottish sites.

#### Staff attitudes and beliefs

*Not ‘on board’:* Overt resistance to the required change of practice was identified at some sites. Staff who were not trial-trained were often unhelpful in supporting VEM provision. Various reasons were given for this including:

*Probably their skill level, and just their confidence…[PT]*

*People were quite resistant to changing their practice… [NS]*

*…some nurses who may not see it as part of their role… [PT]*

*…some people are reluctant to do perceived more work [PT]*

Where nursing staff were not ‘on board’ it meant additional work for the physiotherapists and appeared to contribute to a few sites dropping out of the trial.

*Beliefs about roles and capabilities:* At some sites delays to mobilisation were experienced if nurses waited for a physiotherapist to attend. This was less of a problem where nurses were skilled and confident in patient handling and where there was a trusting interdisciplinary culture around mobilisation.

However a few physiotherapists believed that their discipline should maintain control of mobilisations (especially for patients with more severe stroke), and expressed concerns that risks were increased and clinical reasoning skills could be lost through interdisciplinary, protocolled mobility interventions.

*… a therapy assistant do[ing] a lot of the intervention, potentially is not actually going to help them create positive neuroplastic change. It might be actually potentially reinforcing bad habits…[PT]*

*Beliefs about consequences:* Notable differences existed between sites’ beliefs about the consequences of VEM, which may reflect local beliefs or cultures. Many staff in Australia and New Zealand assumed a positive treatment effect from VEM before the trial results were known. Numerous examples were cited of patients in the intervention arm who had made unexpected recoveries. In contrast, some staff at Scottish sites were less optimistic and in fact worried that early mobilisation of patients with severe stroke would give *‘…that kind of false hope’* [NS], especially to family.

#### Patients’ barriers

*Acuity, instability and complexity*: A proportion of patients were only able to fulfil part of the VEM protocol due to acute health issues, medical instability or complexity. These problems were more common early after stroke, as in this example:

*…he was in rapid AF for a couple of days and was obviously too unstable to participate [PT]*

*Severity of stroke:* Drowsiness and reduced cognition in some patients made VEM delivery more difficult, especially early after stroke. Interestingly, staff opinion varied with regards to how stroke severity influenced their ability to provide frequent mobilisation. Patients with more severe stroke required two or three staff to assist mobilisation which often challenged staff resources. Patient autonomy and family assistance was not an option for these patients with severe stroke. In contrast, staff at other sites found people with more severe stroke easier to manage due to established systems of interdisciplinary practice, and the lower frequency mobilisation required which was often closer to their usual practice.

*[For more severe strokes]… because it was a combined physio and nursing …we didn’t really have to do that much extra [PT]*

Patients with milder strokes were challenging for several staff due to the high frequency VEM required. However, other sites found milder strokes easier to manage because they had adapted their usual practices and encouraged patient autonomy and/or family involvement. One PT described how she would encourage patient’s independence:

*It would be ‘The expectation for today is…that you should be sitting up for all of your meals. When your relatives leave the ward, you should try to walk with them to the entrance’ [PT]*

*Fatigue:* Staff reported that fatigue was the most commonly reported problem for patients, sometimes contributing to refusal to mobilise.

*…with everything that happens in an acute setting, they become very, very fatigued and…because a lot of our patients are elderly it really impacted on them… sometimes the patients would just go ‘enough’… [NS]*

Fatigue was thought to be more often a problem for patients with severe and moderate stroke than milder strokes. A few staff recognised a need for patients to get rest and so modified VEM accordingly. The amount of time mobilising in each session, rather than the frequency of interventions, was reported as problematic for some and needed modification as described by this PT and assistant:

*…the amount and the minutes of doing that would be a problem…so 20 minutes a session or something like that, which some people can’t tolerate [PT]*

*…we restricted our timeframe like we didn’t let them sit out in a chair for too long so we had to cut it short like 40 minutes and that’s it …[PTA]*

*Family anxiety:* Some staff noted family anxiety about the VEM intervention, particularly regarding patients’ fatigue. Some patients’ families believed rest was necessary for recovery, and more important than mobilisation early after stroke.

*The family members were worried because they thought a patient at this acute stage should be in bed resting… [PT]*

Interviewees suggested that differing cultural beliefs about rest and recovery could be a factor.

### Overcoming implementation barriers

Many, but not all, interviewees described successful strategies for implementing the VEM intervention. Several important and interconnected factors were identified as assisting implementation including interdisciplinary teamwork, processes to get staff ‘on board’, and learning to work differently.

#### Teamwork central to success

Sites where staff felt that they had been successful in providing frequent mobilisations spoke of shared interdisciplinary roles with physiotherapists, nurses and other disciplines working closely together through collaboration, negotiation and sharing the workload. Flexible work practices and a willingness to delegate and trust colleagues’ ability to mobilise were important. Mobilisation was ideally considered the responsibility of the whole team, as summed up by one nurse:

*“…truly interdisciplinary stuff…a team approach that recognises that early mobilisation is supported, and therefore we should all modify our practice in whichever way we can, to somehow, it might be in a minimal way, but add to that overall affect. [NS]*

*Communication and coordination:* Staff at several sites reported having high functioning teams. These teams used systematic daily team planning to organise who, when and how mobilisations would occur. Planning was particularly helpful when nurses and all allied health staff involved with trial patients were involved, so that extra mobilisations could be integrated into the patient’s timetable. An example provided for one site was:

*We have a meeting in the morning…our basic structure for the day… discussing with the nurses that if we did this at this time could you do this, you know, could you stand them up to do their teeth, and then… I might get them out of bed, can you get them back into bed at this time… [PT]*

Important verbal communication occurred formally at daily team meetings and nursing shift handovers. Regular informal communication, especially between nurses and physiotherapists, was important for sharing the workload, prompting and trouble-shooting to ensure mobilisations occurred. Some sites used written reminders successfully including message boards on the ward or at patient’s bedside, staff handover sheets, or group emails. Other sites implemented a communications book accessible to trial staff.

#### Getting staff ‘on board’

Many staff were enthusiastic about participating in the trial and were driven by their interest in the research question and intervention. However this enthusiasm was not universal. Almost every interviewee spoke of the importance of spending time and effort getting staff on board or engaged with the new practice.

*Staff education and training:* Staff education and training was a cornerstone to getting staff on board. Ongoing training and support instilled confidence with staff and after some experience with delivering VEM many staff began to embrace the trial and feel an ownership of the protocol. Upskilling nurses to be confident with mobilising patients after stroke was an important strategy to get them involved, and to build interdisciplinary trust. Due to frequent staff turnover, education and training needed to be ongoing to avoid the practice-change losing momentum. Clear VEM protocols and procedures, including summary protocol reminders worn on lanyards, were found helpful by staff.

*Leadership for change:* Implementing VEM required big picture thinking and cultural change, and top down support was important to facilitate this. While practice change was seen as a whole team responsibility, most interviewees spoke of the need for change champions and strong enthusiastic project leaders. Multi-disciplinary leadership or co-leadership by a PT and a nurse was seen as ideal by many, as nurses were more responsive when led by another nurse, as described by one PT:

*…it's helpful to have at least one of those champions being nursing staff …from a physio point of view we're not often here on weekends, we're not here at night time. So I think having a champion from a nursing staff point of view is a big thing [PT]*

Joint leadership also eased the leadership burden, assisted sustainability of the work when one person was away and provided a deeper understanding of the issues, obligations and pressures of the respective disciplines.

#### Working differently

‘*This is what we do here’:* Sites who spoke positively of their ability to implement VEM described a determination to work around organisational barriers and to foster a culture whereby VEM become normalised.

*You just need to get whole-ward involvement that that becomes standard. Like change that mentality that it’s unusual [PT]*

Additional mobilisations became easier where the whole team incorporated mobility into other daily tasks that would not necessarily contain a mobility component. Many sites were innovative and adopted a philosophy of *‘taking every opportunity to mobilise’.* Examples included patients walking part of the way to appointments off the ward, and group exercise sessions. At one site nurses wrote a mobility component into patients’ continence management plans; at other sites there were several examples of allied health disciplines, other than physiotherapists, incorporating mobility into their therapy sessions for example:

*…even if you're a dietician …maybe saying to that person, ‘Well, let's get you sitting out, and we can have a bit of a chat’ [NS]*

*Shifting control:* At sites where staff were able to relinquish some control of their traditional roles and practices, VEM implementation appeared easier. This required physiotherapists trusting nurses to mobilise patients in their absence, and was underpinned by good training and mentorship for nurses.

*…every profession that’s in that unit…has got a responsibility of being mindful of getting that patient out of bed, if not out of bed out of the chair, or do something useful. [PT]*

A further shift in control was seen at several sites where patient and family autonomy was promoted for patients who could cope with this. Strategies to involve patients and their families in early rehabilitation including setting self-mobilisation goals and ‘homework’ tasks, and getting patients to keep activity diaries, all of which were reinforced with education and explanations.

*I actually think the higher level ones were quite engaged in the process. [PT]*

New Zealand sites were particularly mindful of keeping Maori family members informed and involved with the patients’ management.

*Staffing model changes:* In addition to thinking about mobilisation as an interdisciplinary task, some sites found different staffing models useful. Nursing or AH assistants (wardsmen in NZ) played valuable roles in providing additional mobilisations and/or provided second-assistant help to mobilise patients with severe stroke. While some physiotherapy staff found time taken with students a barrier to VEM provision, other units made use of student assistance to provide extra mobilisations.

*Managing fatigue:* Staff’ response to patient fatigue differed. Some described the need to respect fatigue, while others tried to convince patients to mobilise despite it. For patients prone to fatigue, innovative interdisciplinary teams planned their day to work with this, timetabling harder and lighter activities and rests across the day.

## Discussion

Our study contributes new evidence to inform implementation strategies for ASUs and for rehabilitation trials. The findings of our research, qualitatively derived from participants with first-hand experience, provide insight on the challenges of implementing complex new rehabilitation practices within complex settings. We have gleaned information on strategies for overcoming implementation barriers, but have also uncovered areas that require further exploration.

Despite the challenges encountered, many staff were enthusiastic about their participation in the trial and were driven by a fundamental interest in the research question. Participants at many sites described successful participation in the trial, detailing innovative, practical, interdisciplinary team-based ways to ensure the trial intervention was implemented as per protocol. This information will provide guidance to future implementation initiatives. Other sites and individuals however were less enthusiastic about trial participation, and described a greater struggle implementing VEM. Our study helps to unpack some of the reasons for between-site differences in ease of implementation, while raising interesting questions for future research.

It is well established that hospitals, and the units within them, vary in their standards of performance. The systematic review by Taylor et al [21] identified several characteristics of high performing hospitals. Interestingly many of these characteristics (such as interdisciplinary teamwork, building and maintaining a proficient workforce, effective leadership, positive organisational culture, senior management support) are similar to the factors our participants identified for successfully implementing VEM.

Pragmatic rehabilitation trials necessitate behaviour change by individuals, teams and systems at participating sites. Behaviour change is a complex phenomenon [8, 9], and individuals, teams or an organisation’s readiness to change can vary greatly [22]. Although researchers sometimes consider the context (site characteristics) into which they will embed their research, this information is rarely used to select optimal trial sites or to prepare research sites for trial participation. Information is available to identify individual’s or organisation’s readiness to change [22], and there is some evidence that readiness to change can be positively influenced through targeted strategies [22]. Future research could consider such strategies, to prime sites prior to trial start-up.

A strong finding in our study, and which supports other recent rehabilitation trial findings [17], was the importance of highly effective interdisciplinary teamwork. This is perhaps unsurprising as teamwork is recognised as a key feature of good healthcare, and training to improve team functioning improves clinical outcomes [23]. Effective clinical teams have particular qualities and competencies including effective leadership, situation monitoring, mutual support, communication and a commitment to achieving quality outcomes [24, 25]. Teams within ASUs tend to be selected for their clinical expertise, yet high functioning teams require a balance of individuals with various teamwork skills, such as co-ordinators and completer-finishers, to contribute to team goals [26]. Teamwork models such as Belbin’s team role theory have been used for decades to facilitate improvements in business settings [26]. We currently do not know whether the balance of team roles or teamwork competencies in stroke teams may account for some of the between-site variability in rehabilitation implementation.

One team role that was considered very important by our study participants was that of the leader/s. The leaders’ attitudes and personalities were highly influential. Participants highlighted the benefits of discipline-specific leadership and that co-leadership was therefore important in multidisciplinary research. While the AVERT researchers had recognised this and tried to establish co-leadership, success was variable across sites. Currently little is known on how best to select the right trial site leaders, how to prepare them or support them for their challenging roles, or how best to ensure succession planning.

A strength of this study is that data were collected and analysed prior to knowing the results of the AVERT trial. Participants were generally optimistic that the trial would have positive results, and this optimism probably helped sustain staff and researchers to continue in the trial over several years. It is now known that the higher dose VEM intervention resulted in fewer patients overall achieving favourable (no or low disability) outcomes at 3 months post stroke [6]. This raises the importance of ensuring that staff at participating sites see their contribution to research, regardless of their pre-conceived views of the likely effectiveness of the experimental intervention, as important and meaningful. Throughout the AVERT trial, the value of the trial to inform clinical practice in an area of uncertainty and equipoise, was strongly emphasised at all investigator meetings and in newsletters and all correspondence.

Another study strength was that data were collected across three countries. Data from all countries aligned to all major themes, thus strengthening the generalisability of our findings. Although the study was not designed to accurately detect between-country differences, some suspected diversity did emerge. For example only Scottish participants report ward environments as implementation barriers, or were concerned that VEM might raise false hope for families. Only Australian and New Zealand participants perceived a change in usual care over the life of the trial.

Many, but not all, participants in this study described a willingness to facilitate VEM by relinquishing some control, such as physiotherapists enabling nurses to mobilise patients first, or giving patients and families more autonomy with exercises. This paradigm shift is reliant on personal attitude shifts but also adequate coordination, skill development and training to allow autonomy to be trusted. Our finding that increasing autonomy facilitates the delivery of rehabilitation interventions is supported in other stroke rehabilitation studies [17, 27].

Trial treatment fidelity (how carefully staff adhere to treatment protocols) is an essential but challenging part of investigating a complex intervention [17]. Fidelity provides confidence that the intervention was delivered as intended so that outcome results can be correctly attributed to the intervention. In AVERT, we provided detailed trial protocols to treating staff, trained staff in protocol implementation, conducted site initiation sessions to discuss and solve local barriers, provided reminders, decision tools and ongoing phone and online support for queries. Site champions were employed, with co-leadership from a nurse and a physiotherapist. In addition, an external monitor provided feedback to site investigators and the management team on completion of each participant (usual care or VEM) so that deviations in protocol adherence could be quickly addressed. We recommend that trial protocols of complex interventions incorporate an implementation plan with the approach that will be used to achieve fidelity, appropriate measures of fidelity, and how this will be monitored and used to maintain acceptable fidelity standards.

Some interviewees volunteered that the usual care at their site had changed over time. Usual care was not standardised, however, careful trial monitoring of the care and activity provided to patients in the ‘usual care’ and VEM groups was able to track this change to usual care over time, and provide reassurance that significant between-group differences in activity were maintained [6]. While we cannot be certain why usual care changed, it seems more complex than just site-based control group contamination. It can be hypothesised that many staff held an assumption that VEM was effective and that this ‘unconsciously’ influenced usual care over time. There were also external influences, with the recommendation to mobilise early and intensively already appearing in stroke guidelines [28]. Many staff described a site culture of continually striving to improve their standard practice, and participation in a trial may not be able to influence this.

We believe that the site-specific solutions and facilitators staff reported, played a pivotal role in maximising trial fidelity. Despite the comprehensive strategies used in AVERT to ensure trial treatment fidelity, our qualitative process evaluation highlights important challenges that should be further considered in complex intervention trials.

## Conclusion

The AVERT trial, considered a landmark trial in acute stroke rehabilitation, required commitment to delivering an intervention that needed strong collaboration between nurses and physiotherapists and was different to current care models. Many teams succeeded in doing just that. What this qualitative study contributes is unique insights into what factors may be critical to successful trials teams, and as AVERT was a pragmatic trial, success factors to delivering complex intervention in clinical practice.

### Ethics approval and consent to participate

Ethical approval was obtained in Australia, New Zealand and Scotland (see details in Methods section). All participants provided informed consent for participation.

### Consent for publication

All participants provided consent for their de-identified data to be analysed, reported and published.

### Availability of data and materials

The raw data from participants is securely stored at the University of South Australia for a period of seven years. Data applicable to the conclusions of this manuscript are provided in Additional file 1.

## Additional file

Additional file 1: Appendix 1 Supporting quotations. (DOCX 46 kb)

## Abbreviations

ASU: Acute stroke unit
AVERT: A Very Early Rehabilitation Trial (Trial registration ACTRN12606000185561)
PT: Physiotherapist
NS: Nurse
NHMRC: National Health and Medical Research Council (Australia)
UK: United Kingdom
VEM: Very early mobilisation (the AVERT intervention)
