# Supplementary material for: Implementing a complex rehabilitation intervention in a stroke trial: a qualitative process evaluation of AVERT
Source: BMC Med Res Methodol. 2016 May 10;16:52. doi: 10.1186/s12874-016-0156-9 (PMC4862225; doi:10.1186/s12874-016-0156-9)
Supplement: Additional file 1: — Appendix 1 Supporting quotations. (DOCX 46 kb) [file 12874_2016_156_MOESM1_ESM.docx]

**Appendix 1 Supporting quotations**

| **Themes & *Sub-themes*** | **Supporting data examples** |
| --- | --- |
| **Category 1: Staff experience of implementing the trial intervention** | |
| Extra work but rewarding | *Extra work*  “It was time-consuming. There were times when I would need to do extra – see patients in lunch time when I wouldn’t usually do that. [Physio]  “… it is a challenge, like trying to fit that in, do you know, around half eight, half four, so it's basically every hour you've got to try and, and fit that in. And that's quite a lot in a day...not, not necessarily so much a lot for the patient, it's a lot for you going back in and out” [PT/NS focus group]  “It’s a lot of extra work within our normal – compared to standard care.” [Nurse]  “It was really actually challenging to be honest, just the logistics of it all. It’s quite a complex thing to be able to manage…” [Physio]  *Extra work but rewarding*  “… it was an enjoyable experience for all of us, even though it was hard work, it wasn’t easy, it was hard work.” [physio]  “It was time-consuming. There were times when I would need to do extra …see patients in lunch time…It added interest to the workday and defined a new challenge at work. So I found it quite enjoyable” [PT] |
| Team practice changes | “But it really made us work together as a team, and like I say it was a driver for all of us at that point in time, to look at how we all can help each other to achieve the targets.” [Physio]  “Certainly, we probably worked better with the nurses at organising across a day, that this is what would be done to achieve it than we would generally have done. “ [Physio]  “…when it worked well, the team working together and the patients really responding … and the fact that the whole team worked together towards that.” [Physio]  “The first word that comes to my mind is cohesion and that’s what I remember working with [physio] and our other nurses, that you work together…our ward’s changed a lot… before AVERT, you had a culture of, “Oh, we’ll wait for the physio to see what they can do.” You can’t do that with the AVERT trial, you can’t say, “Oh, well I’ll wait,” because you’ve got the goals for the intervention of getting them up, whether you’re a nurse or a physio…So, you worked together. You just didn’t leave it for one discipline to work out.” [Nurse]  *Changes to nursing practice:*  “If they’re quite – a level one patient and they need to be hoisted, say, three times a day, that’s heaps of extra work. You need to sit them up for breakfast, so that’s not something we would usually do.” [nurse]  “…it’s really good getting the nurses involved early, and I think changing the expectation a little bit of nursing care and also making like encouraging the patient to be up and about, which probably isn’t normally the case.” [Physio]  “…for nursing they were very positive about it. Well they could see a benefit for nursing, but it raised the profile of nursing as well, which was a very important thing as well to happen.” [Physio]  *Changes to physio practice:*  “…it gave me a little bit more role for the physiotherapist rather than just making recommendations about where the patient would likely to be discharged to…” [Physio]  “Look, for me I think the participation in AVERT has really made me rethink my practice … thinking about delivering of service and intensity…” [Physio]  “…it challenged me to come up with new exercises for different patients… It added interest to the workday and defined a new challenge at work. So I found it quite enjoyable” [Physio]  “…it was good because, you know, you got to use your skills as a therapist, … So that was rewarding. And yeah, it was good to have that extra patient contact as well. Often working in an acute ward you normally didn't get to do that, spend so much time with them.” [Physio] |
| Changes to usual care | “I think the biggest problem with AVERT was trying to stop early mobilisation happening… when it shouldn’t. It was really hard, and the reason I think was because … early mobilisation was becoming a very important, shall we say… I mean, it was written in all the protocols and the guidelines internationally, so people were saying “oh yeah, early mobilisation, it’s good”’ [Physio]  “So we had so much change, and I think that one of the things that wasn't constant, was what we considered to be our standard care had changed dramatically, from the time of commencing AVERT, to the time of when we eventually finished.” [Nurse]  “I've noticed we have had to pull back over the last, because I've been doing this so long, pull back on what, keep them on track of what is standard still. So that will be - yeah I think some of the, if it is proven to be working, I think some of that change is started to happen and, well not starting to happen, but the impetus for change has started to happen.” [Physio]  “I don't know, I think... we definitely do things quicker than we used to. Do you know, we definitely get people in the stroke unit quicker.” [NS/PT focus group] |
| **Category 2: Barriers to trial implementation** | |
| Team challenges | “I think if you don’t know when you’re showering them or what other things the nurse needs to do that day, you can’t work around that and then you go and try and do your intervention and they’re in the shower but the rest of you day’s blocked, so you’ve kind of lost an hour or two which just means you’re going to miss target for the day.” [Physio]  “I think if you had a whole team, a whole ward team that was on board and that was really interested and really proactive about it, then it would make it significantly easier to do. That has been a difficulty for us” [Physio]  “Q: Did you have a system to coordinate things or did the nurses do their mobilisations and the physios do theirs fairly independently?  A: Yeah, very much independently done.” [Nurse]  “So without the nursing staff assistance with early mobilisation we've - I think we've - probably there's been a greater emphasis on the physio staff being the ones doing the intervention. So I think that's made it harder as well.” [Physio]  “ yeah but for stroke care….yeah we all kind of work in our little professions and that kind of cross profession stuff would be great…………….and the reasons why we do this trying to inform other staff….you obviously have a better plug if you have got evidence there.” [NS/PT focus group]  *Team leadership:*  “…she’s such a good leader who would actually make very good communication with nursing staff, bridge the gap and will make sure that the patient care gets the priority, like at any cost. And her clinical reasoning was so, so sharp and she would actually make sure she achieves the goals…But now unfortunately she’s in community” [Physio Assistant]  “I believe that we may have just wrapped up, just because [name] has been our lead investigator, and she's obviously on leave from clinical duties, so I think it's sort of been wrapped up.” [Nurse]  “Then when I came back…a lot of the patients who the nursing staff used to be very good at getting up and getting moving were often documented as rest in bed till physio review. So I did notice a big difference, most likely because I had that year off and so things changed a lot in that year” [Physio]  “it needed that key person who had a passion for it to drive it, and that’s probably what happened here, that whenever we didn’t have that person, that key person driving it and keep educating staff, and sort of keep checking and screening, that it sort of, from my impression, it fell through then.” [Physio]  *Nurses respond to nursing leaders:*  “It sort of been really physio led… We haven't had good nurse leadership on the ward of the whole AVERT thing. It's been a real challenge.” [Physio]  “Yeah, so she was a nurse. And she was really good at driving it. I did find it challenging when she wasn’t there to get the nurses on board because she would coordinate at times.” [Physio]  “Because as a physio it would be very hard for me to say to a nurse ‘you should be mobilising this patient a number of times’ or ‘did you not realise that the protocol for this is...?’ It then becomes us telling them what to do, and I really don’t like that.” [Physio]  “…she tried her damnedest to get it going and I think it is, it’s not worked particularly well here, absolutely through no fault of [PT leader] and I think if it had only been a physio thing and there weren’t any other... disciplines involved, it would have worked beautifully ... there’s also a leadership issue, erm, with the nurses and I think that’s mainly why it fell down...I don’t know if it wasn’t seen as a priority by the nurses …or they didn’t see where the advantage for them was going to be.” [PT focus group] |
| Staffing challenges | “In my unit I would see that would be a problem insofar as we probably haven’t got the right patient to physio ratio” [Physio]  “There are patients on my list that I’d love to see three or four times a day but I can’t. That’s just not possible and sometimes I don’t even get to see them every day. That’s got nothing to do with what I think is best for them but the time and the staffing that I have available.” [Physio]  “I think our biggest barrier is staffing… this week I have people on leave, people sick and I’ve only got 50 per cent staffing in that area and that’s just a bit of a chronic problem for us.” [Physio]  “Just staffing is a constant issue for our team. If you've got vacancies or sickness and that sort of thing, which we regularly have” [Physio]  “P1: Do you have a, an assistant that you're able to...  P2: No…We don't have any extra staffing for AVERT anymore, not for a long time.” [NS/PT focus group]  *Out of hours staffing:*  “…there is no physio care service to stroke unit at the weekend, for example…So they weren’t getting very early mobilisation in a few cases, if patients were admitted on a Friday night or Saturday morning. A lot of those patients would be not mobilised until assessed by physio, which would have to wait until Monday. So that greatly impacted it.” [Physio]  “…we had to talk a lot with the morning staff members to relay the message over to the night staff who actually didn't work with us so they were not very confident in getting patients up… sometimes the night staff would not get patient up and will just normally hand over the urinals to the patient and stuff like that.” [Physio Assistant]  “…it is difficult on night shift, there’s not a lot of trained night duty staff.” [Nurse]  “So if they come in in the evenings then it could – you know, we may sort of keep them in bed.” [Nurse]  *Caseload demands:*  “So the physios themselves certainly really… did want to engage and do what was required, however, they were constrained by their workload.” [Nurse]  “I physically just couldn’t provide the treatment and see my other patients.” [Physio]  “And I just try and do my best, like getting them, or yeah. Sometimes you could do it, and sometimes you can't. So it just depends. Because you've got other patients to look after as well.” [Nurse]  “A lot of the staff finds the balance between the, “This is just because it’s a study but I’m not seeing my other patients,” the next patient is getting less and they find that really difficult.” [Physio]  “It's just quite challenging sometimes when you've got the caseloads of trying to get the balance to make sure that they're getting the target dosage, but also maintaining care for your other patients and making sure that they don't miss out on their regular input.” [Physio]  *Competing clinical priorities:*  “the acuity of other patients or pressure for discharging patients because the hospital is really full and they're wanting you to prioritise those. So it's having to work in conjunction.” [physio]  “…it really depends on the other patients that you had to look after. “Like, that did sometimes make it difficult… if you had another really unwell patient, like you were focused on them more, so it just depended really. But it wasn't that bad.” [Nurse]  “…if you had other patients that were really prioritised or quite sick sometimes you had to make that time, or sometimes you didn't possibly get that time…Especially with nurses, you've got other patients as well… it’s not always strokes, it can be mixed with other patients that require high care as well.” [Nurse]  “…your AVERT physiotherapist, not only have they got a responsibility for neuro, but they're seeing, perhaps the general population on any acute ward. So there was always that difficulty in getting those extra interventions.” [Nurse]  *Staff turnover:*  “…we have our same challenges in that there’s a high staff turnover in nursing. There’s some challenges in terms of local knowledge and processes and how things actually work. “ [Physio]  “…maintaining staff has been a significant barrier … in the fact that we had people, you know, your nurses move on, timeframes for recruitment of your nursing staff and also pregnancies and all those sorts of things.” [Nurse]  “I think as people came and left it was harder to keep people… the new staff, as they came on, I don’t think… probably weren’t as enthusiastic as the ones from the beginning” [Physio]  “…when they had a consistent physio there it was fantastic, it was really good, everything ran really smoothly, it was really co-ordinated, and then when there wasn’t necessarily a consistent physio …it didn’t work as well…the nurses haven’t been quite sure who was the physio and when they’re around and how to contact them if they need them, things like that.” [Physio] |
| Organisational or workplace barriers |  |
| *The acute model and culture* | “…the acute nurses on the stroke unit don't necessarily have that, well that culture of getting them up, getting them moving, that sort of thing” [Nurse]  “[extra mobilisation is] something we would do on the rehab ward but perhaps not so much on the acute ward, just because of the number of admissions and discharges and just because of the business generally of the ward. “ [Physio]  “I think sometimes – it’s probably a nurse’s catchcry but sometimes just the busyness and the acuity of what would be on the ward at that time” [Nurse]  “Just the sheer fact that they’re in the acute ward that it’s just mainly time restraints” [Nurse]  “There’s obviously your prioritisation system your priority ones are your chest and your discharges.” [Physio]  *Other early tests and procedures:*  “…the patient would have been taken away to a scan … they have access to all their procedures in quite a quick timeframe often within 24 hours, the patient may not be available on the ward. There’s a lot of interruption which is great for rapid management of the medical, surgical issues related to their stroke at the end of the day, but may interfere with them being able to be mobilised” [Physio]  “…if they were having multiple tests off the ward they would be gone for long periods of time… it was sort of hard to get time to get the patients then to go and do their mobilising, because they were either fatigued or busy with other allied health...More so at the very start, because everybody wants to see the patient straight away” [Nurse]  “…logistically quite difficult, especially probably in the first – like the days one to three, when they’re having lots of investigations and then on flexi-monitoring. It can just be logistically difficult” [Physio]  “Well most of the patients that were on AVERT trials were also thrombolysed, so very early mobilisation … we could just get them up post 24 hours post the infusion because that was the protocol.” [Nurse]  “P1: The other thing that influences it is what investigations they are undertaking….obviously they have just been admitted and they have come in the last few days and they have a lot of investigations to get done and sometimes ‘Mrs so and so is going for a scan and she will be going in a trolley so lets not get her upright now lets wait till she has been for a scan and get her up later.’  P2: or we will get her up and put her back to bed.’ [NS/PT focus group] |
| *Barriers to ASU access* | *Delays getting to hospital:*  “But it was hard for us to get patients in within the 24 hours of their stroke. You know, from that because our state’s so big it takes five hours to get [to hospital]” [Physio]  “Capturing them really within that 24-hour window was the single biggest problem that we had. A lot of the strokes that we had present were - even before they came to hospital were outside the recruitment window.” [Physio]  *Delays getting to ASU:*  “…it wasn't as much of a pragmatic trial in that it wasn't taking patients when they first presented to hospital. It was always taking them within that first 24-hour window. As we found that the number of people that don't meet that criteria is huge.” [Physio]  “…if the patients weren’t in the acute stroke unit, it definitely had an impact, if they were in outlier or in emergency, then it would impact when they were mobilised because of the environment” [Physio]  *Outliers and mixed wards:*  “…when the patient was to move out of the stroke unit into the ward area there seemed to be less of a focus and attention on the treatment groups. It was seen that if they moved out of the stroke unit that they were then stable and didn't need the treatment any more” [Physio]  “Another issue that we did have… if the AVERT patient is the most stable patient on the ward they would be transferred to another ward…I'd have to run and see them on the ward but even trickier for the nurses because the nurses on other levels are definitely not AVERT nurses.” [Physio]  “…as part of the changes to our ward, we had other medical patients added to our ward. We used to be just a stroke and neuro ward, but we also take gastroenterology patients on our ward now. ..we've found they were missing out on the rehab side of things.” [Physio] |
| *Competing priorities* | *Organisational changes & conflicting priorities:*  “…we've got heaps of changes all the time... We're always getting emails or a new policy, new protocol. It's like yep, okay.” [Nurse]  “…as part of the changes to our ward…We used to be just a stroke and neuro ward, but we also take gastroenterology patients on our ward now… the gastroenterology patients were requiring a lot of nursing input and acute nursing care and they were generally seen as a higher priority than doing the rehab side of things.” [Physio]  “Then, at one point in time, the ward – we had a number of people who were participating, but then the ward got split. So some units got moved and most of the trained nurses got moved to the other ward.” [Physio]  “There was a lot more focus in general on our ward on assessing the patients and getting the patients discharged, as opposed to providing rehab.” [Physio]  “…there probably is conflict because the change is probably occurring at the same time as demand for improvement across other areas of hospital work… For example… in a hospital accreditation year the expectation is around staff appraisal and being able to show that we’re fully compliant with standards.” [Physio]  *Restrictive policies:*  “our physiotherapist can only do three doubles what we call them or heavy patients per day. So that was put in place because we have multiple work cover claims within physiotherapy and so we needed to do something to ensure that staff weren’t hurting themselves.” [Physio]  “The patients that have for example, have received TPA, they – hospital policy sort of governs that they be resting in bed for twenty-four hours post thrombolysis, so that would be a hospital policy type thing that would be acting as a barrier there.” [Nurse]  “The one sometimes that we do struggle with a little bit is the upper limit for the blood pressure…particularly after a bleed... Because there's a safe limit for AVERT, but it's actually not within what's been individually prescribed and what's in our haemorrhagic/stroke guidelines.” [Physio]  *Competing for frequent mobilisations:*  “We almost found that they would never get the dosage they needed, either because you see they’re away having investigations, appointments, they go off the ward themselves for a coffee or stuff …and then all of a sudden you’d come in and find they were discharged” [Physio]  “The issue arose when …you needed to access them up to nine, ten times a day. That was the main issue there. The patient started to decline and get sick of you coming in and wanting to get them up and going again” [Physio]  “if …we want people to be mobilising or doing an hour of earlier mobilisation, then potentially it could become disruptive with other sessions needed to be done and with speech pathology are wanting to do early intervention” [Speech Pathologist]    “I’m just thinking about OT and Speech and their understanding of it…I know when we have an intervention patient, they find it quite difficult sometimes to find time to see them and sometimes that if that person’s Speech, that they’re not fatigued when can they get a go at them as well” [Physio]  *Inflexible systems:*  “Rest periods sometimes can be a challenge. On our unit there is a rest period…it is quite hard when the room's dark and the family have got the impression that they get to rest for two hours.” [Physio]  “…obviously meals; you don’t want to interrupt the meals. It’s quite important that they eat.” [Physio]  “So, probably the nursing was the most difficult part of it because we really didn't get that the AVERT nurse was necessarily the one who was looking after the client” [Physio]  “We don’t place the AVERT patients with specific AVERT nurses… Because it’s too hard to specifically allocate a nurse who’s AVERT trained with a patient, when we’ve got three other units. We’re not just a dedicated stroke unit.” [Nurse]  “…the physios tended to have a set time in the day that they would come and see stroke patients on the stroke unit, and that time of the day tended to be after lunch… even towards the end of the day…it was simply a suggestion that, well, why don't we have the physios come and see the stroke patients earlier in the day? It's not that they're doing additional work… But that fell on deaf ears, and didn't get off the ground …People were quite resistant to changing their practice” [Nurse]  *Managerial barriers:*  “We probably wouldn’t have had strong leadership from what I’m told on the neuro ward …or even a very strong neurologist, I mean we have lovely neurologists but not ones who’ve been there for years who are perhaps not necessarily driving through change. So I think all of those factors together meant that it was seen as something that you put in the too hard basket” [Physio]  “…it actually, I think, needs departmental support … the physios are employed through department, rather than through the unit themselves… it requires departmental support for you to be able to, you know, within your workload, do this… and it made it difficult within the department.” [Physio]  “Didn't have a high level of engagement or need to involve the managers, although in hindsight it probably would have been better to have a push or top-down approach possibly and for the management to tell - for the management to become real sponsors of the project perhaps a bit more than they were. Yeah, that would have been probably better.” [Physio]  “I think from the department, at the head level, again there was recognition that it had value, there was recognition also that the staff were pretty much at their full capacity. And therefore they, while they said it's great, they didn't necessarily have the power or will to say to their therapists, we want you to consider doing it that way, so that we might get those early interventions under our belt….at that time we didn't have an allied health department head” [Nurse] |
| *Physical environment barriers* | “…access to wheelchairs in particular to have patients out of bed… we don’t stock wheelchairs in the hospital because there is no space” [Physio]  “Environmentally, you know, there's the high-low beds that don't go low enough so that you can put someone's feet on the ground, and when they're sitting on the edge of the bed.” [Physio]  “..access to the equipment because we only have one hoist and there were times where we didn’t have – we had the hoist but not the sling because the sling was off getting laundered” [Nurse]  “…for example, there were people that I was wanting to mobilise the other day, but the slings had gone – to use the standing lifter, I couldn’t do that.” [Physio]  “…if you had a patient who was isolated in a single room for any particular condition, it was a lot harder to mobilise them and keep up with the mobilisations…” [Physio]  “P1: If anything the only thing that would hold it back would be the chairs actually. Yeah.  P2: But yeah, I mean, that's...that would be maybe a reason why you wouldn’t get somebody as early as...” [NS/PT focus group]  “P1: What do you think would help you do this … you know group work apart from staffing?  P2: An area that you could take them to…you know if there was a TV room that they could go and speak to each other or something that you could take them to…just a room that they could go” [NS/PT focus group] |
| Staff attitudes and beliefs |  |
| *Not ‘on board’* | “I think to a degree it came down to targeting the right nurses. I had more success with some rather than others and a lot of it is personality and some people are reluctant to change or do more work, or do perceived more work, whereas others were more than happy to assist and do stuff.” [Physio]  “…there'd be a couple of nurses who have probably worked in the ward for a long time too who are probably slightly resistant to it with regard to how much more work it was going to make for them.” [Physio]  “And I found if I got people out of bed, then the nurses expected me to put them back to bed, that sort of thing. I think you need to have everyone on board otherwise it’s too much on one person to do.” [Physio]  “So I think - yeah, it's like any change program. Just there's some people that will be opposed to change and others welcome it.” [Physio]  “there are some nurses who may not see it as part of their role as such” [Physio]  “… a lot of the people around …well, they're generalists…So they don't necessarily see themselves as a stroke professional, and so getting them to commit to a stroke specific research trial, throughout the whole process has been problematic.” [Nurse]  “You would say ‘They're an AVERT patient. If you could get them up’ …but if they weren't an AVERT nurse they wouldn't, you know, they would not even listen sort of thing.” [Nurse]  “I found that speaking directly to the consultants involved with the patient would be… quite beneficial in that respect, because once you got a decision from the consultant then you didn’t have to pussyfoot around a registrar or resident, who would always be more cautious,” [Physio]  “…hire the people with the right attitude – enthusiasm is the key to success for early mobilisation.” [Physio]  “P1: if it’s more deeply embedded then people would be more accepting...  P2: Yeah, yeah.  P1: ... of taking on board new things.  P2: Yeah” [PT focus group] |
| *Beliefs about roles and capabilities* | *Waiting for the physio:*  “...we'd usually try and get the physios to be the first people to get them up, if it looks like they've got a sort of significant deficit. So if they come in in the evenings then it could – you know, we may sort of keep them in bed” [Nurse A5]  “Mobilising is seen to be more a physio thing …we often get written in the nursing care plan … ‘await physio mobility review’, and we come in the next day and we’d say ‘well hang on, we’ve done mobility training with you…so how come you didn’t mobilise the patient, because they actually look okay’…They say ‘oh they’re walking with a limp so you said not to walk them if they had a limp’, and I said ‘no but they can get out of bed and get on a commode, you can stand them up’” [Physio]  “…a lot of the patients …were often documented as rest in bed till physio review” [Physio]  *Capability built on skill:*  “Q: Can you enlarge on difficulties with the nurses providing it?  A: Probably their skill level, and just their confidence and, you know. Willingness I suppose, to actually, you know, help you as a therapist I think was fine.” [Physio]  “Well also it depends on your training of your nurses as well. If you’ve got like agency staff, they really don’t know quite what to do, and will do nothing. So they won’t even get them up, put them in a chair.” [Physio]  *Maintaining control:*  “If you've got a very challenging patient with quite complex manual handling for example. Then having a healthcare assistant or a therapy assistant do a lot of the intervention, potentially is not actually going to help them create positive neuro plastic change. It might be actually potentially reinforcing bad habits… I would feel that physios would need to have a very active role in promoting it, and making sure that whoever was providing intervention or mobilising the patient, that it was done appropriately” [Physio]  “I’d be looking at case by case and being more individualised with your patients. Just because that early mobility is good and has been found to be good you still need to make sure that’s not just put down as standard. People still have to clinically reason.”[Nurse]  “ I think at the end of the day, it still needs professional judgement on an individual case” [Physio B1]  “…what you don’t want is a recipe. You don’t want to stop people thinking and do their clinical decision-making.” [Physio] |
| *Beliefs about consequences* | *Belief that VEM was effective- Nurses:*  “I think it's a fantastic idea to start them mobilising earlier. You could definitely see improvements in some of them.” [Nurse]  “I think it’s a fabulous intervention that’s typically always got positive results….” [Nurse]  “I personally, think it’s great for the patients. I can see them really benefit from it.” [Nurse]  “Yes, I think it's very good. I think that strokes should have early mobilisation post their stroke. I think it gives them a better outcome, rather than just resting in bed or just lying there… I believe and what I've seen is that, yes, it is - it gives them a much better outcome for while they're in hospital and also a much better outcome for when they go to their rehab.” [Nurse]  *Belief that VEM was effective- Physios:*  “From what I've seen over the three years I've been doing it now the ones that do get very early mobilisation seem to have a faster recovery and a quicker progression towards their normal.” [Physio]  “It was good to see progress. So sometimes when you’d done a little bit of extra with them and you could see them getting better it was easier to go in and do more.” [Physio]  “I think we’ve seen some results for people who’ve made some substantial recoveries that we wouldn’t have expected. So that’s been good to see.” [Physio]  “I would recommend that they actually follow this program, because it is quite -- like it’s quite nicely and safely set for the patient care…its actually best for the patient care.” [ Physio Assistant]  *False hope*  “P4: …everybody looks better when they’re sitting up.  P5: ... so, again, it maybe gives that kind of false hope.  P4: Uh-huh, yeah.” [NS/PT focus group]  “P1: Another thing you have to respect is if they do have a low GCS… then to sit someone out that may give their relatives the wrong impression so we have to… that it is not indicating that they are improving its just a trial period…  P2: Yeah patients always look better when they are sitting up.” [NS/PT focus group]  “P3: Sometimes there's unrealistic families.  P4: Uh huh, and we have, we've had a few, they've seen them up sitting and they think this is...they're cured, and things are getting better and they're not. And they probably won't either, but you have to give them every chance.” [NS/PT focus group] |
| Patients’ barriers |  |
| *Acuity, instability and complexity* | “…sometimes patients when you recruit them, may be completely appropriate fitting all the criteria. Then subsequently have medical issues that come up …if they’re unwell or whatever.” [Physio]  “…if someone came to us and they were, say, MRSA or MRE we had to isolate them in a room…it was a lot harder to mobilise them and keep up with the mobilisations in that respect.” [Physio]  “if they’re sick – you know…medically unwell. For example, we’ve just had a gentleman that – really acutely unwell and we couldn’t get him out. Which is fine, I know.” [Nurse]  “From a medical stability point of view I can only recall one patient where it was tricky because …he was in rapid AF for a couple of days and was obviously too unstable to participant” [Physio]  “Sometimes they had blood pressure problems and heart rate problems, so getting them up can be like a rock and a hard place. You’ve got to get them up but then you don’t want to send them into a rapid heart rate or have the blood pressure go through the roof and have an episode.” [Nurse]  “I had one recently where she was pregnant and she was having a lot of sickness and nausea. So she was just generally not feeling that well and for her, that was a bit of barrier” [Physio]  “If you’ve got, if you’ve got people that are quite unwell then obviously the nurses, that's their priority, you know, so your rehab patients tend to take a wee bit of a back seat…” [NS/PT focus group] |
| *Severity of stroke* | *Severe stroke more challenging than mild stroke:*  “If they’re quite – a level one patient and they need to be hoisted, say, three times a day, that’s heaps of extra work.” [Nurse]  “…the small number that might have had a cognitive deficit, it was problematic because we couldn't employ some of the other strategies… getting assistance to actually follow commands and do the things that we would have liked to have done were limited.” [Nurse]  “Also I think for lower level patients the very early is not always appropriate because they’re often very drowsy and as much as getting people out of bed and getting them upright increases their alertness, it doesn’t always get them alert enough to get a really positive benefit from it I think.” [Physio]  “For all patients that were low mobilisation. So obviously transfers and requiring at least two nurses to move, it was quite difficult if they were in the above standard care category. Just the time constraints and ratios and that sort of thing, but for the patients that were more mobile and more sort of independent, it was easier to get that either standard level of care or above standard level of care.” [Nurse]  “The severe strokes, it’s hard to mobilise them, especially if they were full assist with two people and a hoist. Being a busy ward it was hard to pin down someone on occasion to help you with the lift.” [Nurse]  “Rarely did we get patients good enough that families could help …Usually we ended up with patients far too heavy physically for families to be able to help out” [Physio]  “The ones that could mobilise themselves were so much more easier.” [Nurse]  “…for the higher level patients it was a bit easier because I mean we would just generally work it in and around their goal setting anyway… it would be, you know, ‘The expectation for today is that you’re going to go here for the shower, about this time and that you should be sitting up for all of your meals. When your relatives leave the ward, you should try to walk with them to the entrance of the ward’” [Physio]  *Mild stroke more challenging than severe stroke:*  “Ones and twos were normally okay. I found that easier, I know it was longer and they're heavier but I found that easier to deliver because it was more collaborative sometimes with the nurses.” [Physio]  “The ones that are low level, I felt like we met VEM criteria without really doing a huge amount more than we would normally do because it was a combined physio and nursing and I think the entire load between us and the nurses we didn’t really have to do that much extra” [Physio]  “I found the threes and fours sometimes a little bit trickier just because the number of interventions is higher.” [Physio]  “We found the harder was when we got to the level 4 patients who needed to be mobilised up to eight times a day, that was where it was very different from what we'd be doing…it was the ones that really needed just that little bit of assistance or nudging to go though that were the struggle ones.” [Physio] |
| *Fatigue* | “Sometimes if they went to physio, of course, they're going to get very, very tired, so the mobilisation sometimes for nursing staff wasn't - sometimes they need to have more sleep or more rest” [Nurse]  “…fatigue of the patient as well. Having to provide multiple interventions throughout the day and that sometimes fatigue would limit participation in terms of a patient feeling too fatigued later in the day and declining to participate for some of the intervention” [Physio]  “…with everything that happens in an acute setting, they become very, very fatigued and we find and because a lot of our patients are elderly it really impacted on them… sometimes the patients would just go ‘enough’… ” [Nurse]  “The only thing is obviously patient fatigue in the level ones and twos towards the end of the day…When you're going for your fifth or sixth session they're the ones that are rating a bit higher on their Borg scales and are requiring a little bit more encouragement to participate...I did have a few refusals in there despite my best efforts” [Physio]  “[mobilise] as soon as possible but depends on other medical problems or, there are so many, I mean if they have been sitting about in accident and emergency you know they are just too tired” [NS/PT focus group]  *Fatigue noticeable for severe strokes:*  “Fatigue is probably one of the harder things to work around when patients are that low level and exhausted after the first time you see them. Then you're trying to see them another three or four times.” [Physio]  “…the level one patient was still very acute and very heavy. They were absolutely buggered at the end of the day they were very tired. Sometimes, like I said, I was just sitting with them and hardly did  anything because I thought it wasn’t reasonable and found it wasn’t benefiting anymore.” [Physio]  “…the physio would put the intervention in… but the amount and the minutes of doing that would be a problem…so 20 minutes a session or something like that, which some people can’t tolerate. Younger people can, but older people can’t” [Physio]  “…sometimes the more affected ones were drowsier so they wouldn’t last the time. So, the physio session…it was tiring for them. [Nurse] |
| *Family anxiety* | *Belief that rest is better:*  “I do recall family members saying, “No, they’re too tired. They can’t do treatment today”. And I have a feeling one of them might have even just been in the standard treatment group” [Physio C1]  “…the family members were worried because they thought a patient at this acute stage should be in bed resting.” [Physio]  “…one particular incident the patient had a haemorrhage stroke for example, and the family were really like thinking oh it’s a brain haemorrhage and …the patient himself should not be doing the mobilisation and should be rested… I tried to convince the family but they were not too keen or they were really nervous about it …and the physio then …came into the session and explained them that this is what we are doing, and it is not meant to harm” [Physio assistant]  “…the family members were worried because they thought a patient at this acute stage should be in bed resting… For the ones that wanted the reassurance, I pretty much ran through how do we during the exercise time, how do I ensure safety, and make sure that nurse and the therapy aid does. So we talked about blood pressure is monitored, we talk about the blood sugars, and the fact that using the bulk scale to make sure that patients are not overly exhausted” [Physio]  “…sometimes family members actually became an issue, in terms of them not wanting you to do too much with their family member.” [Physio]  *Believing cultural influence:*  “I think it’s linked so some of the ethnicities that we deal with, with some of their kind of health beliefs. In some cultures, you find are a bit more difficult than others to get them moving early” [Physio]  Some of the issues that we had is that we had quite a culturally diverse patient population. That at times put a little bit of a spanner in the works I guess in terms of early mobilisation, obviously for stroke patients that already do have a deficit and trying to communicate with that at times is a little bit difficult” [Physio] |
| **Category 3: Overcoming implementation barriers** | |
| Teamwork central to success | *Inter-disciplinary teamwork:*  “So for everybody to understand and be working on it together, more like an interdisciplinary plan so that it’s not always just reliant on the physio. Once the physio’s done the initial assessment, if someone needs supervision then the dietician can supervise somebody getting out of bed and encourage the same.” [Speech Pathologist]  “I think any health care professional could or should be doing it. I don't think that it should fall to one person. I think that caring for individuals with stroke can become very hard, if you choose to work in silos.” [Physio]  “…truly interdisciplinary stuff, rather than still doing what your practice says you should do only. So a team approach that recognises the fact that early mobilisation is supported, and therefore we should all modify our practice in whichever way we can, to somehow, it might be in a minimal way, but add to that overall affect.” [Nurse]  “…it just depends on what the level of disability is…if it's someone with sort of a mild deficit, then I think anyone should be able to provide that early mobilisation. But obviously sort of your more complex and heavily debilitated person, or affected person…I think probably a more sort of physio/nursing combined mobilisation would probably be more appropriate.” [Nurse]  “P1: Athough we are very multidisciplinary I sometimes think that perhaps we are…it could be….more blurring of the rolse…..interdisciplinary is that the word I am looking for?  P2: How do you think you could get a more interdisciplinary feel?  P3: I think in a way it has already started, therapists…involved more with all the care, rather than bringing a nurse to do the task, already we are seeing the blurring and I think given more of a blurring of the roles…so if there was a quiet moment why you know two nurses could do …say walking a patient up the ward… that 5 minute walk done 3 times a day not necessarily by the physio might be the difference in getting that man home before Christmas.” [NS/PT focus group]  *Trusting colleagues to mobilise:*  “…it’s everybody, every profession that’s in that unit, work in that unit, has got a responsibility of being mindful of getting that patient out of bed if not out of bed out of the chair or do something useful.” [Physio]  “We certainly had the occupational therapists on occasions, depending on how much they were willing to “cross that line”. That they’re flex.” [Physio]  “Well I think all members of the team…I think it’s what’s safe, and obviously, but the occupational therapist as well because I think in terms of mobilisation can be included in things like…when they’re doing showering, dressing, even just doing cognitive function if they need to walk a patient, rather than take them on a wheelchair” [Physio] |
| *Communication and coordination* | *Daily team planning:*  “So we will normally meet with them first thing in the morning at 7:30 and set up a plan for the day, particularly for the lower level patients. We'll work with them to work out when the patient will have a shower, what they'll do after a rest. So actually how they will meet their targets.” [Physio]  “Yes we have a handover with all of allied health and we have a handover every morning and then we have a multidisciplinary meetings three times a week for the Stroke Unit and any discharges, we talk to the nurse in charge or the stroke nurse practitioner throughout the day because the turnover is pretty quick.” [Physio]  “We have a meeting in the morning, a journey board meeting. So, you know, our basic structure for the day… otherwise it would be directly, you know, discussing with the nurses that if we did this at this time could you do this, you know, could you stand them up to do their teeth, and then they need to get back into bed at this time so that, like I might get them out of bed, can you get them back into bed at this time, and then manage to get back out here.” [Physio]  “We plan really early, so we plan as a physio team. And depending on their levels really and what else is going on, we have a daily plan. We have morning catch up with the nurses and help them plan…sometimes they need to fit in with us. Sometimes we need to fit in with their plans for the day…. Planning helps” [Physio]  “So the physios and the nurses will step aside from the patient and from non-AVERT team members to talk about what the plan is for the day, usually at the beginning of the day. “ [Nurse]  “We have a meeting in the morning, a journey board meeting…in the mornings we would plan the day. So, from a time perspective…So a lot of it was sort of about thinking and working out what times people would do things” [Physio]  *Timetabling:*  “…try and work around not having it clash with other investigations, or other OT assessments sort of thing. I guess there's going to be little bit of planning involved and just have a think about, you know, ‘I'm on this afternoon, I have got to try and get this person out as much as I can, how am I going to work it through’” [Nurse]  “I think timetabling works… and I think it is easy in an acute setting to think that it doesn’t work, because I think it can feel like there are so many other factors that are involved with that person, but I think actually by doing AVERT, we’ve proved that you can do it” [Physio]  “We almost ended up scheduling I guess appointment times. We started calling them appointment times so that they became a little bit more I guess not ownership but there was an agreed time between therapists, staff and the patient and including their family as well so that they all had a better idea. So it literally was an A4 piece of paper that we pop up at the start of the day which helped quite a lot. We sort of treated it like appointment time rather than you know we’ll see you twice this afternoon, we tried to schedule some times which I think helped” [Physio]  *Informal communication, reminders, troubleshooting:*  “…attending handovers and things like that was very important. So yeah, sitting in on the nursing handover and chiming in about what to do for the patients, that was a good strategy.” [Physio]  “It’s just reminding the staff, checking, you know, throughout the day how we’re going. Nothing extra, I suppose. Just reminding the staff, encouraging them, helping out myself when I’m not necessarily clinical.” [Nurse]  “but also trying to pre-empt, if there was going to be anything that would act as a barrier. So for example, trying to find out early if the patient was going to go for a scan or something or some sort of an investigation so that we could take that into factor and still try and meet target.” [Physio]  “…we just would say, “Look, this person has to get up. We have to get so many moves in,” and you just used communication and a plan for that day… So, it was mainly the verbal handover at the end of each shift.” [Nurse]  “…the physiotherapists liaised with the nurses that were looking after the patients. So, they may say for example, ‘I'm going to treat patient X this morning for two sessions of ten minutes. Can you do two sessions of ten minutes either after lunch or at afternoon teatime?’ So, they would usually work together and just have an informal communication plan” [Physio]  “…if I’m providing my own intervention, I still might go up and go ‘Okay how are you going?’ to the nurses...I think it’s really important, I think it’s one of the primary things. It doesn’t work if you’re doing it independently.” [Physio]  “P1: Good communication  P2: Talking to each other talking to relatives  P3: Yeah in all…across the board…backwards and forwards you know all directions” [NS/PT focus group]  *Written strategies:*  “So it was almost like a rehab planner was used for them and that was given to the nurse and to – sometimes it was given to the patient as well and it was given to the therapy assistant…we just would write a plan and the nurse would have that plan.” [Physio]  “Then we’d just leave a sheet on the front of a patient’s section where you record their observations and they just had to document that and that was sort of the communication process.” [Physio]  “…we devised a system of getting the nursing staff who were looking after the patients just to write down everything they did.’ [Nurse]  “…on our communication board, next to the patient’s bed space …Then normally what would happen at first thing in the mornings, the physio that was co-ordinating that patient would set out a daily timetable for them.” [Physio]  “Sometimes we would literally give them the recording sheet where they record what they’re doing, we would actually give it to them to keep in their pockets so they would act as a bit of a visual reminder.” [Physio] |
| Getting staff ‘on board’ | “Our experience was overall quite good in terms of the early intervention with the stroke patients. Because, we had quite an engaged multidisciplinary team, we found that most of the staff were on board.” [Physio]  “You’d need to have – a whole ward team that was on board and that was really interested and really proactive about it, then it would make it significantly easier to do.” [Physio]  “It has to be the actual floor staff that are looking after the patients. Because they’re the ones – you’ve got to make sure that they’re involved because they’re the ones that are actually going to be doing the work, predominantly. You need to have them on board for it to be successful.” [Nurse]  “Definitely to focus on nursing staff involvement. I don't think it can be done without the nursing staff getting on board and getting the early mobilisation training with the nursing staff.” [Physio]  “I think getting the team, so getting everyone on board is really important, and that's not just at ward level…to be sort of wider spread than just at ward level.” [Nurse] |
| *Staff education and training* | *Education to get team on board:*  “Definitely that everybody in the stroke’s team understands the reason behind it, the research …So I guess education to everybody in the team and communication and then as new staff come on, continuing that education” [Speech Pathologist]  “I don't think it can be done without the nursing staff getting on board and getting the early mobilisation training with the nursing staff.” [Physio]  “I think just getting the right people involved, getting the education behind the staff as well to raise their awareness, and rationale as to why it's a positive thing and sort of work from there.” [Nurse]  “the whole team, I think the doctors, the nurses and all the physios need to be educated, what it is, why we’re doing it and that it doesn’t necessarily mean more work, and if it does it’s about patient care rather than anything else. Also education to the families…” [Physio]  *Education for confidence and trust:*  “…the first thing to my mind there was training. You know, more on techniques on how to mobilise patients, manual handling training and just education, you know, on what the benefits are.” [Physio]  “So it's really, the education's quite a big thing and really normalising it.” [Physio]  “But if it was proven to be the best case scenario, you’d need to have that in the hospital protocol so it was standard and everyone was good to go on it.” [Physio]  “I would recommend following the guidelines that tell us what a stroke unit should be… a lot of it revolves around education and awareness about what best stroke practice is” [Nurse]  *Ongoing education:*  “We're running in-services every rotation for the nursing staff so they're handling stuff isn't too bad. For the intensity of intervention we probably need to have in services and training.” [Physio]  “It’s a constant education and constant reporting back to them that is required.” [Physio] |
| *Leadership for change* | “…ideally it would be fantastic if there was …one of the very senior nurses, so the clinical nurses, so the ones who are still obviously working on the floor. I found that they can be quite influential as well, because on our ward particularly, they're quite experienced, but very dynamic people, and very patient focussed. So they can really motivate the rest of the staff, so that would be fantastic” [Physio]  “You need people who actually tell them all the time again, “come on keep going, you have to do it again” you need some strong leadership I think.” [Physio]  “…I would say for the team leader of the acute stroke unit, either be the doctor or…the nursing unit manager, that that person would be best to have some rehab focus to appreciate the importance of rehab in a stroke unit.” [Physio]  “…just getting a team of enthusiastic people together to make sure that it is rolled out. So most of our projects here they are very multidisciplinary because everyone is involved with the care of the patient.” [Nurse]  “P1: You know, I think you'd need somebody, I don't know, it would be more a champion thing, you need somebody kind of focusing everybody to lead it I think….  P2: So it would be quite nice for maybe an individual, if they were happy to embrace this idea of very early mobilisation, had it shown to be effective, to actually coordinate it. And remind people as well, "Okay, right, we should be getting these patients up." [NS/PT focus group]  *Physio-Nurse co-leadership:*  “I think it's useful probably to have two people, like a like a lead from therapies and a lead from nursing, to actually make sure that it's happening and then use them for governance planning and quality things to review progress” [Physio]  “I think you definitely need the physios to be primarily responsible but definitely to get your NUMs on board too. They would be instrumental in getting those nurses to accept it as regular practice.” [Physio]  *Nurses respond to nurse leaders:*  “I definitely think there needs to be a champion on the ward. I think there probably needs to be a few and I think it's helpful to have at least one of those champions being nursing staff as well, because I think from a physio point of view we're not often here on weekends, we're not here at night time. So I think having a champion from a nursing staff point of view is a big thing.” [Physio]  “…you're going to have to champion it on the floor… to really be promoting and championing it amongst the nursing staff to really get it going…ideally it would be fantastic if there was a senior nurse, like the CNS, or the clinical nurse specialist…” [Physio]  “…she was a nurse and she was really good at driving it. I did find it challenging when she wasn’t there to get the nurses on board because she would coordinate at times.” [Physio]  “…it needed someone to drive it, and I think that fell on me initially, to actually, you know sort of drive, make sure things were happening, follow up with the nursing staff about have they done their mobilisations…so maybe they relied on me to much…Whether I did make them a little bit too reliant on me in the end” [Physio] |
| Working differently | *Working around organisational barriers:*  “At one institution that I've worked at we do have a rule that only lets you do three doubles a day. If a patient was a level one or a level two sometimes instead of doing three mobilisations that would involve, for example, doing stands with a level two patient, then a fourth and fifth one might have to be a sit as a single and then some stuff in the bed.” [Physio]  “The hospital enforced a rest period but we’re lucky that the ward was happy for us still to go in and access patients for physio in that time. So that wasn’t an issue.’ [Physio]  “…if I come back to the manual handling sort of issue, it would require additional equipment, machinery, you know, like hoists, standing lifters, than we currently have available… So there would need to be investment from that point of view.” [Physio]  “…lower level seating so for getting people up safely, we just don’t prop them up for something that might be mal-adaptive…Seating cushions like pressure care” [Physio]  “... but we have, we’ve changed our prioritisation system for bank holidays and for the first time acute strokes have gone down on the bank holiday list.” [NS/PT focus group]  *Mobilisations becoming part of daily care routine:*  “…even if you're a dietician for example, there's nothing to say you shouldn't be maybe saying to that person, ‘Well, let's get you sitting out, and we can have a bit of a chat’” [Nurse]  “we should endeavour to have that knowledge and the nursing practice of saying, ‘Yes, we will’ - instead of the patient sitting in the bed for meals, we say, ‘No, we can get the patient out for meals’, or ‘We will get the patient out for a shower, not have a wash in the bed’. So all those things…This is all early mobilisation… all those little things that a nurse can do.” [Nurse]  “So, pragmatically it's about inserting it into your daily plan and just taking any opportunity that we can to, you know, to take someone for a walk.” [Nurse] |
| *‘This is what we do here’* | “You know it's normal that from day one that they’ll actually get up for every meal and you have a shower and things every day. If they're seeing that as normal rather than - yeah, not related to the trial, ‘this is what we do here’” [Physio]  “You just need to get whole ward involvement that that becomes standard. Like change that mentality that it’s unusual, ‘That’s what happens for everyone. This is what they do.’ You really need to get everyone on-board to have that work” [Physio]  “…even now as a nurse, you just say, "No, this is important. This is important, because while they're in hospital, if us nurses don't get them out of bed or let them - or sit them out in a chair, then they're just going to be in the bed". So even these little things, as I said, out for meals, or go on the commode instead of just wash when they can. All these little things add up to big things.” [Nurse]  “So most of it I take that’s just how it is, that’s work.” [Physio] |
| *Shifting control* | *Shifting control of mobilisation:*  “…even if you're a dietician for example, there's nothing to say you shouldn't be maybe saying to that person, ‘Well, let's get you sitting out, and we can have a bit of a chat’” [Nurse]  “we should endeavour to have that knowledge and the nursing practice of saying, ‘Yes, we will’ - instead of the patient sitting in the bed for meals, we say, ‘No, we can get the patient out for meals’, or ‘We will get the patient out for a shower, not have a wash in the bed’. So all those things…This is all early mobilisation… all those little things that a nurse can do.” [Nurse]  “…it’s everybody, every profession that’s in that unit, work in that unit, has got a responsibility of being mindful of getting that patient out of bed if not out of bed out of the chair, or do something useful.” [Physio]    “We certainly had the occupational therapists [mobilise] on occasions, depending on how much they were willing to “cross that line”. That they’re flex.” [Physio]  “But it's not my job to be the only person that can get them up out of bed, because the nurses are more than qualified to be able to make a clinical judgement about somebody, and not keep them in just because I've not been here on the weekend and then I phone in sick on the Monday morning and suddenly Monday afternoon everybody goes, "Why's everybody still in bed?" Do you know what I mean, that just doesn't actually make any sense, does it?” [PT in PT/NS focus group]  *Increasing patients’ & families’ autonomy:*  “…family can be an important aspect of the care. So I guess trying to incorporate them into treatment sessions was something which sometimes is preferable” [Physio]  “And then giving them a role to play in their family or whoever it was; in their recovery as well, so they can then feel a part of the treatment as well. So, that often helped us quite a lot and particularly they were quite often concerned if their relative didn't speak English as the first language…” [Physio]  “I think families should get involved, but I think in selected cases I think it’s what’s safe” [Physio]  *Information & education for patient & families:*  Perhaps some information as well for patients and families. I think that’s key. Not just educating staff, but patients and their families as well.” [Physio]  “…more education both the staff…and also the family. They could even make a little brochure or something explaining why it is so important to the family in simple language” [Physio]  “I also think we need to educate the population who have actually had a stroke in the family. Most people who come to hospital just expect that they will be in bed and that they actually need to sleep and rest. Their perception of how to recover is something we have to address.” [Physio]  *Cultural influence of family involvement:*  “I think it’s linked so some of the ethnicities that we deal with, with some of their kind of health beliefs. In some cultures, you find are a bit more difficult than others to get them moving early” [Physio B3]  “…we would consider, I guess, the different view of health that the Maori have and that includes the spiritual side.” [Physio]  “So understanding what's important to them, what family members are important. They'll often have a lot bigger family with them when you're doing the interventions, but you can actually utilise that. They'll often tend to help you as well.” [Physio]  “The engagement of the family is a big thing for Maori and that actually can make physical rehab easy because you’ve got more people motivating that person to do it.” [Physio]  “We try and make them as much a part of the process with their family—not more so than other cultures but it’s definitely something that we make sure is kind of at the forefront for them because we know that’s important” [Physio] |
| *Staffing model changes* | “I think using more of the nursing health care assistants, which they do tend to use a little bit in the trial, but probably could utilise a lot more.” [Physio]  “I think that we should be pursuing how we can use family, carers, that kind of thing, aides perhaps more, to deliver interventions in higher dosage. Like, that's going to be important for implementing it widely across the board.” [Physio]  “With the more heavy people here we now have more stable wardsmen people on the floor who help getting people up for every meal as well as with the two assist. I think that’s a way in how they can be utilised.” [Physio] |
| *Dealing with fatigue* | *Respecting fatigue:*  “Then it's just the challenge of getting the timing right and keeping, so that the patients aren't too tired, but also keeping the family happy that they don't feel that you're exhausting their relative.” [Physio]  “If there were any concerns, in terms of …the level of fatigue, those sorts of things, if there were concerns around that, well we just acted accordingly. It wasn't a case of well, we must, we must, because. It was a case of, well, we back off and we do what we can. And then you put in the reason why a mobilisation didn't occur when perhaps it should have” [Nurse]  “…we restricted our timeframe like we didn’t let them sit out in a chair for too long so we had to cut it short like 40 minutes and that’s it and we all get together to put him back in bed” [Physio Assistant]  *Convincing to mobilise despite fatigue:*  “…usually you just got, ‘I’m too tired’ and, you know, they’d enrolled in the study. …You’d tell them it was in their best interest and sometimes could convince them but other times they were just not willing.” [Physio]  *Planning around fatigue:*  “…so I not only looked at the nursing practice, I will look at my other allied health colleagues, people like the OTs I will look at what they do and how I could incorporate the mobilisations into their sessions. As well as because the way how the mobilisations need to be structured, the patient needs to have plenty of rest. So I also look at their downtime with the social worker, when they are chatting to the social worker, I put that down as a rest time.” [Nurse]  “we also tried to blend that in with a few less challenging, so say something in sitting activities, but that would still be like a bit of a mobilisation, so unsupported sitting for example. So the person felt like they were having a little bit of a rest but they were still getting a mobilisation.” [Physio]  “We would be down there very early in the morning first thing. Working out the number of interventions needed for the level where they’re at. Then liaising with the nurse… trying to work out basically a plan, or when physio is going to see them or OT is going to see them. When nurses are going to do their end in terms of mobilising the patient as well. Because we found if we don't do that, they don't get the rest time they need in between interventions…” [Physio]  “That [fatique] did raise its head as an issue at times. Because, we worked quite closely together as a multidisciplinary team, we were usually aware of when the patients were better than at other times. So, for example some of our patients may have performed better in the morning and others were better at performing in the afternoon. So, as a multidisciplinary team we worked together on that…So, at times it did play a factor. I wouldn't say that it was a limiting factor. It was a barrier, but one that we were able to not work around, but work together to overcome.’ [Physio]  “ I do remember the stroke patients, they get very tired so you had to negotiate, ‘Okay, if I get them out for breakfast, give them a shower, maybe they can have a lay down and there’s another movement, getting him up later on in the day.’ And then you could also incorporate it into an evening shift, so give them a rest, get them up for dinner” [ Nurse]  “…they get very tired so you had to negotiate, “Okay, if I get them out for breakfast, give them a shower, maybe they can have a lay down and there’s another movement, getting him up later on in the day.” And then you could also incorporate it into an evening shift, so give them a rest, get them up for dinner.” [Nurse] |
